# C-REACTIVE PROTEIN AND BRAIN MORPHOLOGY

**DOI:** 10.1093/geroni/igac059.1748

**Published:** 2022-12-20

**Authors:** Rahul Pearce, Lisa Eyler, Tyler Bell, Jeremy Elman, Christine Fennema-Notestine, Donald Hagler, Carol Franz, William Kremen

**Affiliations:** University of California San Diego, La Jolla, California, United States; University of California San Diego, La Jolla, California, United States; University of California, San Diego, La Jolla, California, United States; University of California San Diego, La Jolla, California, United States; University of California San Diego, San Diego, California, United States; University of California San Diego, La Jolla, California, United States; University of California San Diego, La Jolla, California, United States; University of California San Diego, La Jolla, California, United States

## Abstract

The process by which aging leads to increased risk for Alzheimer’s disease and dementia is not entirely understood, but one hypothesized contributor is the occurrence of low-grade inflammation in older age. In this study, we examined how peripheral levels of C-reactive protein (CRP), a measure of systemic inflammation, relate to brain structure and diffusion in a group of older adult men. After excluding twenty-seven participants for confounding medical conditions, we analyzed a final sample of 426 community-dwelling men from the Vietnam Era Twin Study of Aging, who were assessed at average age 68 for plasma levels of CRP and underwent structural and diffusion brain imaging. Linear mixed models adjusting for family relatedness, age, medical morbidity, and BMI examined associations of CRP with whole brain volume, whole gray and white matter volumes, global fractional anisotropy (FA), and global mean diffusivity (MD). Higher CRP was related to lower whole brain volume (β = -.13, p = .006), including lower whole white matter volume (β = -.22, p <.001) but not whole gray matter volume (p = .08). Higher CRP was related to lower global FA (β = -.51, p = .012) but not global MD (p = .203). Regionally, a relationship of higher CRP to lower FA was found in the anterior thalamic radiation (β = -.51, p = .010), which is implicated in a variety of higher order cognitive processes. These results suggest a link between peripheral inflammation and lower white matter integrity in older adult men, the implications of which for cognitive aging and dementia should be further explored.

